# Risk Factors in Patients Who Had Prior Renal or Liver Transplant Undergoing Primary Total Hip Arthroplasty

**DOI:** 10.3390/jcm14103486

**Published:** 2025-05-16

**Authors:** Vikram S. Gill, Sayi P. Boddu, Elie Mansour, Bassam G. Abu Jawdeh, Muhammad Ali Khan, Alyssa McGary, Henry Clarke, Mark Spangehl, Matthew P. Abdel, Cameron K. Ledford, Joshua S. Bingham

**Affiliations:** 1 Mayo Clinic in Arizona, Scottsdale, AZ 85259, USA; 2 Division of Nephrology and Hypertension, Mayo Clinic Arizona, 5777 E. Mayo Blvd., Phoenix, AZ 85054, USA; abujawdeh.bassam@mayo.edu; 3 Mayo Clinic in Minnesota, Rochester, MN 55905, USA; abdel.matthew@mayo.edu; 4 Mayo Clinic in Florida, Jacksonville, FL 32224, USA

**Keywords:** total hip arthroplasty, liver transplantation, kidney transplantation, survival analysis, postoperative complications

## Abstract

**Background:** Solid organ transplant (SOT) recipients are living longer and, consequently, more of them require elective total hip arthroplasty (THA) to restore mobility and improve quality of life. Because these patients are chronically immunosuppressed and often burdened by multiple comorbidities, their peri-operative risk profile may differ substantially from that of the general THA population. This study aimed to evaluate risk factors associated with acute medical and surgical complications, implant survivorship, and overall mortality in patients with a history of SOT who underwent THA. **Methods:** A total of 173 THA procedures were reviewed in patients with previous SOT. Among them, 64 had undergone liver transplantation (LT), 83 had received renal transplants (RT), and 26 had experienced more than one type of organ transplant (MT). Kaplan–Meier survival analysis was employed to estimate median survival. Complications were examined using univariate analysis through mixed-effects logistic regression, while Cox regression was utilized to assess mortality risk. The median follow-up period extended to 99 months. **Results:** The proportion of patients experiencing at least one acute medical event was 27% in the LT group, 33% in the RT group, and 38% in the MT group, with no statistically significant difference between groups (*p* = 0.5). American Society of Anesthesiologists Class (ASA) 4 (Odds Ratio (OR) = 28; *p* = 0.006) and treatment with bisphosphonates (OR = 2.25; *p* = 0.03) were associated with higher risk of acute medical complications. Increased age at the time of SOT was linked to a reduced likelihood of surgical complications (OR = 0.94, *p* = 0.008), as was older age at the time of undergoing THA (OR = 0.92, *p* = 0.001). The observed rates of reoperation and implant revision were 3% and 1%, respectively. The estimated patient survivorship rates at 1, 5, and 10 years were 98.6, 82, and 58.4%, respectively. Older age at SOT (Hazard Ratio (HR) = 1.06, *p* < 0.001), at THA (HR = 1.08, *p* < 0.001), ASA 4 at THA (HR = 7.57, *p* = 0.02), and atrial fibrillation (AFib) (HR = 3.13, *p* = 0.02) were associated with higher mortality. **Conclusions:** ASA 4 and bisphosphonates were associated with a higher risk of acute medical complications, whereas older age was associated with lower surgical complications. Additionally, older age, ASA 4, and AFib were associated with higher mortality.

## 1. Introduction

Solid organ transplantation (SOT) prevalence has been steadily increasing across the United States, exceeding 25,000 renal and 9500 liver transplantations in 2022 [1]. Additionally, the prognosis for individuals who have received organ transplants has been enhanced by recent progress in surgical methods, perioperative management, and immunosuppressive therapies [2]. As a result, 5-year survival rates for renal transplant (RT) and liver transplant (LT) patients aged at least 65 years are 72% and 77%, respectively [3]. With increased survival in the elderly population, more patients with SOT may develop primary hip osteoarthritis, or secondary osteoarthritis following femoral head avascular necrosis, and subsequently become a candidate for total hip arthroplasty (THA) [4]. Furthermore, transplant patients may be predisposed to requiring THA, as prior research has shown that patients with prior RT underwent THA 5–8 times more commonly than the general population [5].

Although SOT markedly decreases mortality across numerous health conditions, THA is primarily aimed at enhancing patient quality of life through pain relief and improved mobility [6,7]. Among individuals with a history of SOT, 82% to 100% of those undergoing THA report favorable or excellent clinical outcomes [8]. Nonetheless, despite these benefits, postoperative complication rates in this population have been documented to reach as high as 50% [8]. Such risks may be attributed to underlying medical conditions, common comorbidities, such as secondary hyperparathyroidism or vitamin deficiencies, or to the use of high-dose, chronic immunosuppressive medications [9]. These factors may contribute to a heightened risk of infection, bone degradation, and fractures among individuals receiving total joint arthroplasty (TJA).

Large registry- and claims-database studies encompassing >6 million primary total hip arthroplasties (THAs) show that four mechanisms dominate the contemporary revision landscape: aseptic loosening (≈35%), prosthetic joint infection (PJI, 18–27%), dislocation/instability (≈16%), and periprosthetic fracture (≈11%) [10]. Although PJI complicates only 1–3% of primary THAs, infected cases double the episode-of-care cost and, even after staged revision, achieve infection-free survival rates that frequently fall below 80% [11,12]. By contrast, aseptic loosening remains the principal mode of late (>10-year) failure [13].

Among these mechanisms, PJI warrants special emphasis because its pathobiology directly intersects the immunosuppressed state common to transplant recipients. Upon microbial invasion, innate immune cells such as neutrophils and macrophages are rapidly recruited and release pro-inflammatory cytokines like IL-6 and TNF-α, which amplify the local inflammatory response [14,15,16]. Moreover, the formation of bacterial biofilms on implant surfaces can hinder immune cell access and dampen antimicrobial activity, leading to persistent infection [17]. This dysregulated immune environment often results in implant loosening, osteolysis, and failure of the prosthetic joint [18,19].

However, the existing literature lacks comprehensive analysis of the particular risk factors that contribute to elevated complication and mortality rates following THA in patients with a history of SOT. Furthermore, the specific impact of various immunosuppressive agents on outcomes in this distinct patient group remains insufficiently understood. This investigation aimed to identify risk factors linked to medical and surgical complications, implant longevity, and overall mortality in a contemporary, multi-institutional cohort of SOT recipients undergoing primary THA.

## 2. Materials and Methods

### 2.1. Study Design and Setting

**Figure 1:**
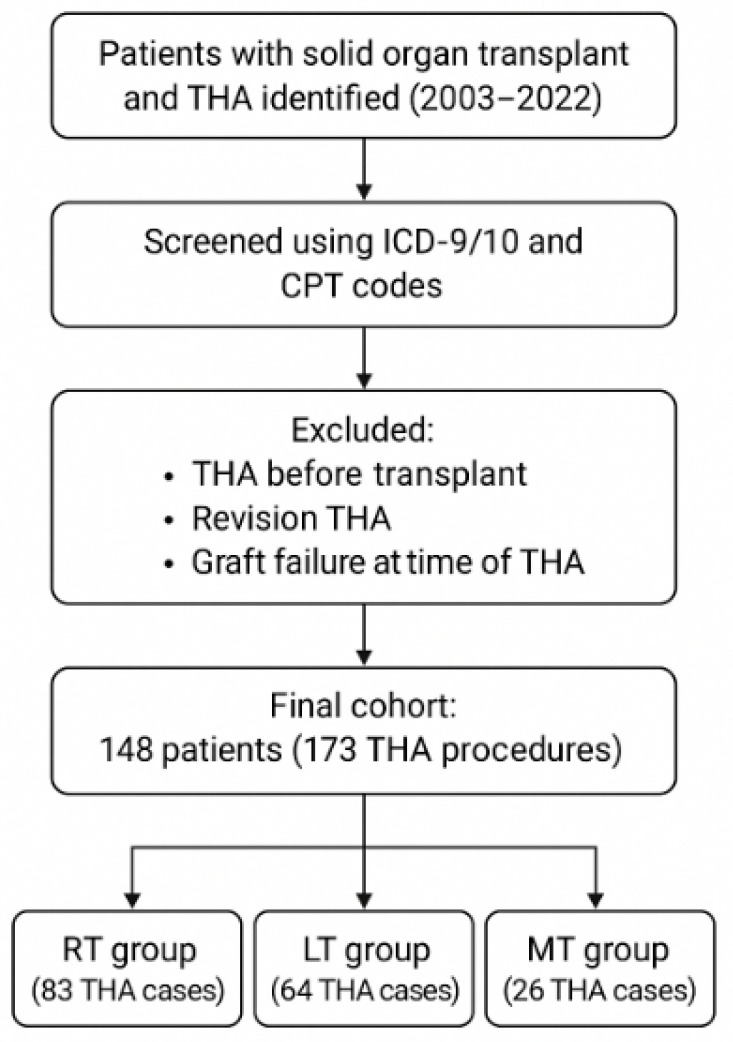
Cohort selection flow diagram. ICD-9/10 = International Classification of Diseases, 9th/10th Revision; CPT = Current Procedural Terminology; RT = renal transplant; LT = liver transplant; MT = multiple transplants.

Patients who had received either a single RT or LT prior to undergoing primary THA between January 2003 and September 2022 were retrospectively identified across all major Mayo Clinic sites, including Jacksonville, Rochester, and Arizona. Identification was performed using the International Classification of Diseases, 9th and 10th Revisions (ICD-9/10), along with Current Procedural Terminology (CPT) codes. An additional cohort consisted of individuals who had received multiple organ transplants (e.g., heart, kidney, liver) or underwent repeat transplantation, categorized as having multiple transplants (MT). Patients were excluded if they had received THA before their transplant, underwent revision THA, or had graft failure at the time of THA. Follow-up extended until the most recent clinical evaluation or death. Institutional review board approval was obtained prior to study initiation. Figure 1 summarizes the cohort selection.

### 2.2. Data Collection

For all included individuals, data were collected on any postoperative medical or surgical complications, any reoperations or revision THA procedures, and the recorded date of death. Surgical complications were defined as those directly associated with the operative procedure or implant, whereas medical complications encompassed all other events. Complications occurring within 90 days of the index arthroplasty were considered acute, while those occurring thereafter, including chronic periprosthetic joint infections, were categorized as late. Reoperations included any surgical procedures related to the index THA, and revision surgeries specifically referred to the replacement of one or more components of the original implant. To investigate risk factors associated with complications, implant longevity, and patient survival, a range of variables were extracted, namely: demographic characteristics, body mass index (BMI), and the American Society of Anesthesiologists (ASA) physical status classification at both transplantation and THA. Additional information included the type of organ donor (living or deceased), the time elapsed between transplantation and THA, fixation method of the implant (cemented vs. uncemented), and comorbid conditions such as hypertension, diabetes, hyperlipidemia, coronary artery disease, atrial fibrillation, and heart failure. The most recent T-score from a Dual-energy X-ray Absorptiometry (DEXA) scan before THA was also recorded for each patient, as documented across all three major Mayo Clinic sites (Rochester, Jacksonville, and Arizona). Data regarding induction therapy administered before transplant surgery, the immunosuppressive regimen used post-transplant, and the use of bisphosphonates or vitamin D supplementation were obtained via a detailed manual chart review.

### 2.3. Data Analyses

Mean and standard deviation (SD) were used to describe continuous variables, while categorical variables were reported as frequency and percentage. Differences across RT, LT, and MT groups were assessed using Kruskal–Wallis rank sum tests for continuous data and Fisher’s exact tests for categorical data. Kaplan–Meier analysis was applied to estimate median overall survival. Univariate analysis involved mixed-effects logistic regression models for identifying associations with any acute medical complication, surgical complication, or reoperation/revision, and Cox regressions were used to analyze mortality. To account for patients undergoing more than one procedure, mixed-effects models were used. Statistical significance was defined as a *p*-value < 0.05. All analyses were conducted using R version 4.1.2 (R Foundation for Statistical Computing, Vienna, Austria).

## 3. Results

### 3.1. Cohort Characteristics

A total of 148 patients, accounting for 173 THAs, were included in the analysis. The mean age at the time of THA was 64 years (ranging from 30 to 89), while the average age at the time of SOT was 59 years (range: 26 to 78). The cohort had a mean BMI of 29.7 (range: 19–48), and 43% were female. Individuals in the MT category tended to be younger at both transplant and THA. Racial distribution showed 94% of patients were White, including those of Hispanic ethnicity, 5% identified as Black, and 1% were from other racial backgrounds. The procedures included 83 THAs following RT, 64 after LT, and 26 in patients with MT. Among these, 67% of RT were from living donors, compared to 13% in the LT group. Most implants (77%) were uncemented, with the fixation approach left to the surgeon’s clinical judgment. At the time of surgery, 90% of patients were classified as ASA class III or higher, indicating significant systemic illness. Hyperlipidemia was significantly more prevalent in RT recipients compared to LT recipients (*p* < 0.001) (Table 1). All patients were on one or more maintenance immunosuppressive agents, including tacrolimus, mycophenolate, sirolimus, everolimus, azathioprine, belatacept, cyclosporine, and/or corticosteroids. Tacrolimus was the most frequently used agent in both RT and LT recipients (94%), followed by corticosteroids, prescribed in 95% of RT and 84% of LT patients. Mycophenolate and corticosteroids were used more commonly in RT patients (*p* < 0.001 and *p* = 0.04, respectively), whereas azathioprine and cyclosporine were significantly more frequent in LT patients (*p* = 0.002 and *p* = 0.02, respectively) (Table 2).

### 3.2. Complications and Risk Factors

In total, 33% of RT, 27% of LT, and 38% of MT patients experienced at least one acute medical complication following THA, though these rates did not significantly differ among the groups. Surgical complications were reported in 6% of RT, 6% of LT, and 19% of MT patients after THA, without statistically meaningful variation across groups. Comparisons of acute medical, acute surgical, and chronic surgical complication rates following THA revealed no significant differences between RT, LT, and MT cohorts.

The most frequently observed acute medical complications of clinical significance in RT, LT, and MT patients were anemia (16%, 17%, and 23%), kidney injury (4%, 3%, and 4%), pneumonia (4%, 3%, and 4%), AFib (1%, 6%, and 0%), and urinary tract infection (5%, 3%, and 0%), respectively.

Major acute surgical complications included wound infection or dehiscence (2%, 2%, and 12%) and periprosthetic fracture PPF (0%, 2%, and 0%) in RT, LT, and MT patients, respectively.

Among late surgical complications, periprosthetic joint infection (PJI) was significantly observed in 1% of RT, 0% of LT, and 4% of MT patients, while dislocation occurred in 2%, 0%, and 4%, and PPF was reported in 0%, 3%, and 0%, respectively. Two cases of late PJI were documented (1%), involving one patient with a single RT and another with two previous RTs. No early hip dislocations were noted, whereas three late dislocations occurred, two in patients with RT (2%) and one in a patient with two prior RTs (4%).

Intraoperative PPF occurred in one hip with prior LT (2%) treated with cerclage wiring during the surgery. Late PPF occurred in two hips (3%) with prior LT at 19- and 42 months post-THA; both fractures were Vancouver A-G, treated conservatively with touch weight bearing. Of note, all three patients with PPF had a prior LT, and their THA was performed at age 53, 54, and 56 years old, respectively. None of the patients with prior renal or multiple organ transplants had PPF. Table 3 summarizes the acute medical and acute and chronic surgical complications.

An ASA 4 at the time of THA (Odds Ratio (OR) = 28, 95% Confidence Interval (CI): 2.63, 298; *p* = 0.006) was associated with higher risk of any acute medical complication compared to an ASA 2. Moreover, treatment with bisphosphonates (OR = 2.25, 95% CI: 1.06, 4.76; *p* = 0.03) was also associated with a higher risk of any acute medical complication.

Furthermore, an older age at the time of SOT (OR = 0.94, 95% CI: 0.9, 0.98; *p* = 0.008), and at time of THA (OR = 0.92, 95% CI: 0.88, 0.97; *p* = 0.001) were associated with a lower risk of any surgical complication. None of the immunosuppressants were associated with a higher risk of any medical or surgical complication.

It is noteworthy that the type of implant fixation (cemented vs. uncemented) was not associated with a higher risk of PPF. This may be due to the low occurrences of PPF, as there was insufficient statistical power to detect this potential association. Nevertheless, the 3 cases of PPF (1 early and 2 late) occurred in uncemented THA (3 PPF/133 uncemented THA (2%)).

### 3.3. Reoperations and Revisions

The total reoperation rate was 3%, with rates of 4%, 0%, and 8% observed in patients with prior RT, LT, and MT, respectively, following THA. Revision procedures were required in 1% overall, distributed as 1% in RT, 0% in LT, and 4% in MT patients. Because of the limited number of events, the analysis lacked sufficient statistical power to identify significant differences in reoperation or revision rates across the various SOT groups or to evaluate associations with fixation method (cemented vs. uncemented). Notably, reoperation was needed in three uncemented cases (2%) and in two cemented cases (5%).

Specifically, two hips with previous RT and one with prior MT underwent reoperation without implant replacement. One patient with RT underwent incision and drainage (I and D) for wound dehiscence within one month of their THA. The other hip with prior RT had closed reduction after dislocation 29 months after THA. The one hip with prior MT had incision and drainage (I and D) for superficial wound infection with *Serratia marcescens*, within one month of THA.

There was one hip with prior RT that required revision. This hip was treated with two-stage revision for PJI with *Streptococcus mitis* 21 months after THA. Another hip with prior MT (two kidneys) required a two-stage revision for treatment of PJI with *Streptococcus viridans* 33 months after THA (Table 4).

### 3.4. Patient Survivorship

**Figure 2:**
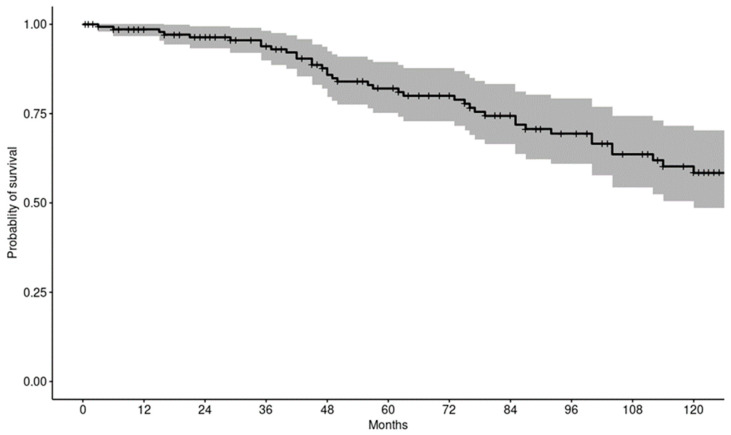
Kaplan–Meier curve depicting overall patient survival probability.

The combined estimated patient survival following THA in individuals with prior SOT was 98.6% at 1 year, 82% at 5 years, and 58.4% at 10 years (Figure 2).

**Figure 3:**
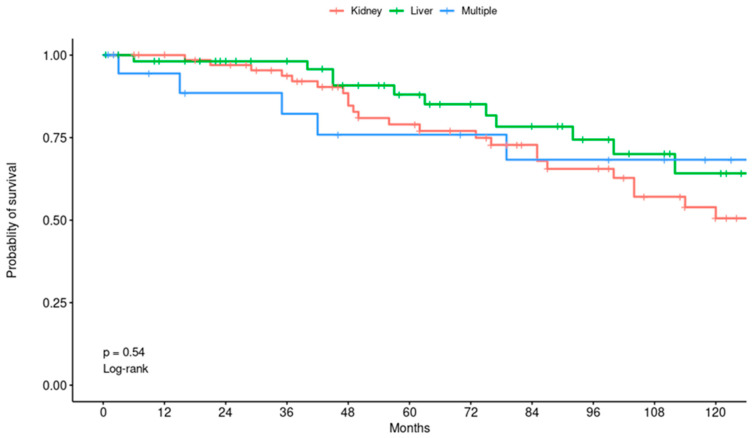
Kaplan–Meier curve showing patient survival probability stratified by transplant type.

Within the subgroups, those with prior RT demonstrated survival rates of 100% at 1 year, 79% at 5 years, and 51% at 10 years post-THA. For LT recipients, survival was 98% at 1 year, 88% at 5 years, and 64% at 10 years following THA. Patients in the MT group showed 1-, 5-, and 10-year survival rates of 94%, 76%, and 68%, respectively (Figure 3).

There was one death within 3 months following THA in a patient who had a prior MT secondary to a severe pneumonia. There were no statistically significant differences in patient survival rates between the three groups. Notably, older age at transplantation (Hazard Ratio (HR) = 1.06, 95% CI: 1.03, 1.09; *p* < 0.001), and older age at THA (HR = 1.08, 95% CI: 1.04, 1.12; *p* < 0.001) were associated with lower survival. An ASA 4 at the time of THA was associated with lower survival compared to an ASA 2 (HR = 7.57, 95% CI: 1.44, 39.7; *p* = 0.017). Also, AFib was associated with lower survival following THA (HR = 3.13, CI: 1.22, 8; *p* = 0.017).

When analyzing survival following THA for patients who had a prior LT alone, a longer interval since LT was associated with better survival (HR = 1.01, 95% CI: 1, 1.02; *p* = 0.05), while a higher level of creatinine (HR = 4.62, 95% CI: 1.56, 13.6; *p* = 0.006) and cyclosporine as maintenance immunosuppressant therapy (HR = 6.8, 95% CI: 1.9, 24.4; *p* = 0.003) were associated with worse survival.

## 4. Discussion

### 4.1. Study Context and Novelty

To the best of our knowledge, this represents the largest single-institution study examining predictors of medical and surgical complications in patients undergoing THA after prior SOT. All patient records were manually reviewed, helping to avoid common limitations associated with large-scale database studies. It is important to acknowledge that a subset of these patients was included in a previous analysis conducted by our group. That earlier investigation included 136 THAs performed between 2000 and 2013 and focused solely on patient and implant survivorship [20]. In contrast, the current study expands the cohort to include additional patients treated after 2013, incorporates updated immunosuppressive regimens, and evaluates comorbid conditions and immunosuppressive therapies not previously analyzed.

### 4.2. Patient Demographics and Donor Characteristics

The average age of SOT recipients undergoing primary THA was 64 years, which closely aligns with the general population’s mean age of 65.7 years for primary THA [7]. Notably, patients with prior MT tended to be younger than those who had received a single organ transplant. A striking lack of racial diversity was evident in the cohort, with over 94% of patients identifying as White or Hispanic. This raises the need for further investigation into equitable access to both SOT and joint arthroplasty.

In terms of donor source, 67% of RT cases involved living donors, whereas over 87% of LT cases were from deceased donors. Our cohort had a higher representation of living donor RTs than national averages, likely due to one institutional site serving as a global center for living kidney donation. Conversely, the predominance of deceased donor livers is consistent with national patterns [1].

### 4.3. Comorbidities

Hyperlipidemia was observed more frequently among RT patients in comparison to those with LT. Given that end-stage renal disease often coexists with diabetes and hypertension, it is not surprising that metabolic disorders like dyslipidemia were more common in RT recipients [21]. In contrast, end-stage liver disease is most frequently caused by alcoholic cirrhosis, hepatitis C, autoimmune conditions, or idiopathic factors. Except for non-alcoholic fatty liver disease, these conditions are generally not linked with metabolic abnormalities [22]. Nevertheless, hyperlipidemia did not emerge as an independent predictor of either medical or surgical complications following THA in SOT patients.

### 4.4. Complications

Although patients with RT and LT exhibited a higher incidence of acute medical complications following THA, the majority of these events were classified as minor. Specifically, acute medical complications occurred in 33% of THAs among RT recipients, 27% among LT recipients, and 38% among those with MT. This was in accordance with other reports. Ledford et al. [8] reported a medical complication rate of 29% in 55 THAs performed in prior SOT patients. Klement et al. [23], comparing THAs with and without prior SOT from the Medicare database between 2005 and 2011, found more frequent medical complications in THAs with prior transplant at 30 days postoperatively. In a more recent meta-analysis performed by Kim et al. [24], the authors compared the rates of complications following primary THA between prior SOT patients and a control group. Pneumonia (2.5 vs. 0.9%) and acute kidney injury (14 vs. 3.4%) were the only two statistically significant medical complications encountered more frequently in the prior SOT patients.

In this study, the incidence of acute medical complications following THA did not significantly differ between patients with a history of RT versus LT. In contrast, Duplantier et al. [25] reported that individuals with prior RT had an OR 6.8 times greater for developing complications after THA or TKA compared to those with prior LT. Similarly, Douglas et al. [26], noted a high complication rate in RT recipients undergoing THA; however, complication rates were still lower than those seen in patients receiving dialysis for end-stage renal disease. Patients on dialysis experienced increased 90-day readmission rates and a higher incidence of revision procedures at 2 years, compared to RT recipients [26].

Despite the elevated risk of acute medical complications often reported in prior SOT patients undergoing THA, the rate of acute and chronic PJI observed in this study (0% and 1%, respectively) was consistent with that reported in the general population [27]. Both PJI cases occurred in patients with a history of RT, including one who had undergone repeat kidney transplantation. Similarly, the incidence of late hip dislocation was low, with only three cases (2%), all in patients with RT or repeated kidney transplants. Bradford et al. [28] found a 5- to 8-fold increase in dislocation rates among RT recipients compared to the general population following THA. Further research involving a larger database could be valuable in exploring whether PJI and dislocation occur more frequently in patients with prior RT compared to those with prior LT.

Similarly, both early and late PPF were infrequent in this cohort, each occurring at a rate of 1%, and exclusively observed in LT patients with uncemented femoral stems. The relatively low incidence of fractures seen in this and other recent studies may be attributed to reduced glucocorticoid dosing and quicker tapering protocols following transplantation. Due to the limited number of PPF cases (n = 3), statistical evaluation of associations with T-score, vitamin D use, or bisphosphonate therapy was not feasible. Interestingly, although RT patients are generally considered at greater risk for compromised bone health due to high-turnover bone disease from end-stage renal disease, the mean T-score for RT patients (−1.1) did not differ significantly from that of LT patients (−0.7), and no increased incidence of PPF was observed in the RT group. Again, a large database study to detect association between PPF and patients who had a prior LT undergoing THA versus RT patients will help determine if this association exists.

Aseptic loosening has been a historical concern following THA in prior RT due to poor bone quality [29]. In the series by Chalmers et al. [20] analyzing THA in prior SOT, only 1.5% revision rate was reported for femoral aseptic loosening, while no cases of acetabular aseptic loosening were noted. Interestingly, no cases of aseptic loosening were observed in our series.

Contradictory results exist in the literature concerning surgical complications in THA with prior SOT. Klement et al. [23] found that, at final follow-up, four of the seven recorded surgical complications were more frequent in THA patients who had a prior SOT, compared to THA without prior transplant. The complications included PJI (OR = 1.6), dislocation (OR = 1.4), mechanical complication (OR = 1.3), and need for irrigation and debridement (OR = 1.9). Interestingly, PPF and need for revision were not significantly increased in their SOT cohort. It is noteworthy that, in their series, RT patients had the highest rate of surgical complications. Agarwal et al. [30] conducted a comparison of 2-year surgical complication rates in patients undergoing primary THA, stratified by the presence or absence of prior SOT. Identified within the PearlDiver database, 3103 patients with primary THA and prior SOT were compared to 6196 patients with primary THA without previous SOT. PJI, dislocation, and aseptic loosening were not significantly different between SOT and non-SOT groups undergoing THAs.

This study offers a distinct contribution by identifying predictors of complications in THA recipients with a history of SOT. A higher comorbidity score (ASA 4) and bisphosphonate treatment were associated with higher acute medical complications following THA in patients with prior SOT. SOT patients who have more comorbidities are expected to have higher medical complications after a major surgery. Khatod et al. [31] found that the use of bisphosphonates was associated with a higher risk of PPF after THA in younger patients, but the effect of bisphosphonate on medical complications following THA was not previously studied. Moreover, older age at the time of SOT and THA was associated with a reduced risk of surgical complications. The lower demand of patients at an older age and being less active may explain the lower risk of surgical complications following THA.

### 4.5. Reoperations and Revisions

In this analysis, the overall reoperation rate following THA in patients with prior SOT was 3%, while the revision rate was 1%, with a median follow-up duration of 99 months. Owing to the infrequency of these events, the study lacked sufficient power to assess differences in reoperation or revision outcomes between RT and LT recipients. The revision rate observed in this cohort aligns with the 2% revision rate at 7-year follow-up reported for combined THA device constructs in the general population [7]. Additionally, PJI and joint dislocation were the leading causes of revision in this series, mirroring the primary indications for revision surgery following primary THA in broader populations [7]. The revision rate noted in our study is lower than the rate reported by Chalmers et al. [20]. These authors reported a revision rate of 6.6% (9 patients) in patients undergoing primary THA with prior SOT. Douglas et al. [32] reviewed multiple databases to identify primary THAs from 2010 to 2019 in LT recipients. None of the medical, surgical, or revision rates were statistically different between the LT recipients and the non-transplant patients. The authors stated that their results are aligned with the previous literature suggesting the same complication profile for THAs performed in LT vs. non-transplant group.

### 4.6. Patient Survival

Within our cohort, a single case of mortality was recorded within 90 days post-THA. Kim et al. [24] reported a significantly elevated 90-day mortality rate in the SOT group compared to controls undergoing THA, with an OR of 2.02 (*p* = 0.04). Chalmers et al. [20] reviewed patient survivorship of primary THA patients with prior SOT. In their series, patients had survival rates of 82 and 78% at 5 years with prior RT and LT undergoing THA, respectively. Likewise, in our series, high patient survival rates were observed following THA, 79 and 88% at 5 years in prior RT and LT patients, respectively. Older age at SOT and at THA, ASA 4 versus 2, and AFib were all associated with lower patient survival rates following THA in SOT patients. Older patients and patients with higher comorbidities are expected to have a higher mortality risk following THA. Additionally, Reynolds et al. [33] found that patients who have pre-existing AFib undergoing total joint arthroplasty have more perioperative complications, which might predispose these patients to higher mortality risk.

### 4.7. Limitations

This single-center, retrospective study relied on ICD-9/10 and CPT codes for case identification, so miscoding could have excluded or misclassified patients. Because only 148 recipients (173 hips) were available, event counts were insufficient for stable multivariable logistic or Cox models; we therefore report univariate mixed-effects results, leaving residual confounding factors. The small cemented-implant subgroup (~23% of hips) and the absence of a matched non-transplant cohort further limited subgroup and comparative analyses. Detailed longitudinal data were often missing, and the fixation method was chosen by surgeon preference, introducing potential selection bias. Collectively, these constraints reduce statistical power and temper causal inferences. Multicenter, prospective studies with larger samples and a full covariate capture are needed to validate these findings.

## 5. Conclusions

Despite inherent limitations, this study underscores important insights into THA outcomes among SOT recipients. While the incidence of acute medical complications was comparable to previously published data, the use of modern perioperative optimization strategies and updated immunosuppressive regimens appear to have contributed to a decline in surgical complication rates. Notably, the cohort demonstrated low occurrences of PJI, PPF, aseptic loosening, and wound-related issues. Also, we found that ASA 4 and bisphosphonate were associated with higher risk of acute medical complications following THA in SOT patients. In addition, older age at the time of SOT and THA was associated with a lower risk of surgical complications. Furthermore, older age, AFib, and ASA 4 were associated with lower survival following THA in patients who had prior SOT. Overall, these findings can assist surgeons in counseling patients with prior SOT and support shared decision-making regarding the risks and expectations of undergoing THA.

## Figures and Tables

**Table 1:** Demographic characteristics at the time of THA. *N* represents the number of procedures rather than individual patients. A total of 25 SOT recipients underwent bilateral THAs. THA: total hip arthroplasty, BMI: body mass index, ASA: American Society of Anesthesiologists Class, SOT: solid organ transplant, Cr: serum creatinine.

Type of Transplant	Kidney (N = 83)	Liver (N = 64)	Multiple (N = 26)	Total (N = 173)	*p* Value
**Age at Time of THA**					0.011 ^1^
Mean (range)	65.1 (30–80)	64.9 (47–78)	57.6 (35–89)	63.9 (30–89)	
**BMI at Time of THA**					0.666 ^1^
Mean (range)	29.7 (19.1–43.2)	30.3 (19.1–48.2)	28.6 (20.0–34.9)	29.7 (19.1–48.2)	
**ASA Score at Time of THA**					0.352 ^2^
2	11 (14%)	4 (7%)	2 (8%)	17 (10%)	
3	66 (82%)	51 (85%)	20 (80%)	137 (83%)	
4	3 (4%)	5 (8%)	3 (12%)	11 (7%)	
**Interval from SOT (months)**					0.217 ^1^
Mean (range)	63.4 (5–255)	71.3 (0–252)	92.9 (4–281)	70.7 (0–281)	
**Cemented (Y/N)**					0.805 ^2^
No	62 (75%)	50 (78%)	21 (81%)	133 (77%)	
Yes	21 (25%)	14 (22%)	5 (19%)	40 (23%)	
**Preoperative Cr**					0.038 ^1^
Mean (range)	1.6 (0.8–6.2)	1.3 (0.7–3.1)	1.7 (0.8–3.7)	1.5 (0.7–6.2)	

^1^ Kruskal–Wallis rank sum test. ^2^ Fisher’s Exact Test for Count Data.

**Table 2:** Induction therapies and maintenance immunosuppression.

Type of Transplant	Kidney (N = 83)	Liver (N = 64)	Multiple (N = 26)	Total (N = 173)	*p* Value
**Induction Therapy**					<0.001 ^1^
Thymoglobulin	38 (46%)	0 (0%)	14 (54%)	52 (30%)	
Alemtuzumab	15 (18%)	0 (0%)	0 (0%)	15 (9%)	
Basiliximab	24 (29%)	13 (20%)	5 (19%)	42 (24%)	
Daclizumab	3 (4%)	2 (3%)	0 (0%)	5 (3%)	
Corticosteroids	3 (4%)	45 (70%)	5 (19%)	53 (31%)	
None **Maintenance Therapy**	0 (0%)	4 (6%)	2 (8%)	6 (3%)	
Tacrolimus	78 (94%)	60 (94%)	24 (92%)	162 (94%)	0.915 ^1^
Mycophenolate	79 (95%)	36 (56%)	24 (92%)	139 (80%)	<0.001 ^1^
Sirolimus	9 (11%)	7 (11%)	6 (23%)	22 (13%)	0.259 ^1^
Everolimus	0 (0%)	1 (2%)	2 (8%)	3 (2%)	0.030 ^1^
Azathioprine	0 (0%)	7 (11%)	5 (19%)	12 (7%)	<0.001 ^1^
Belatacept	4 (5%)	0 (0%)	0 (0%)	4 (2%)	0.210 ^1^
Cyclosporine	1 (1%)	7 (11%)	5 (19%)	13 (8%)	0.005 ^1^
Ongoing Corticosteroid	79 (95%)	54 (84%)	26 (100%)	159 (92%)	0.021 ^1^

^1^ Fisher’s Exact Test for Count Data with simulated *p*-value (based on 2000 replicates).

**Table 3:** Rates of medical and surgical complications.

Type of Transplant	Kidney (N = 83)	Liver (N = 64)	Multiple (N = 26)	Total (N = 173)	*p* Value
**Acute Medical Complications**					
DVT/Thrombophlebitis	1 (1%)	1 (2%)	0 (0%)	2 (1%)	1.000 ^1^
Anemia	13 (16%)	11 (17%)	6 (23%)	30 (17%)	0.657 ^1^
Kidney Injury	3 (4%)	2 (3%)	1 (4%)	6 (3%)	1.000 ^1^
PE	1 (1%)	1 (2%)	0 (0%)	2 (1%)	1.000 ^1^
AFib or Flutter	1 (1%)	4 (6%)	0 (0%)	5 (3%)	0.187 ^1^
Pneumonia/Lung Injury	3 (4%)	2 (3%)	1 (4%)	6 (3%)	1.000 ^1^
Pancreatitis	0 (0%)	0 (0%)	1 (4%)	1 (1%)	0.150 ^1^
Sepsis/Shock	1 (1%)	0 (0%)	0 (0%)	1 (1%)	1.000 ^1^
Electrolyte Imbalance	1 (1%)	0 (0%)	1 (4%)	2 (1%)	0.414 ^1^
MI/Unstable Angina	1 (1%)	0 (0%)	1 (4%)	2 (1%)	0.414 ^1^
Seizure	1 (1%)	0 (0%)	0 (0%)	1 (1%)	1.000 ^1^
Stroke	1(1%)	0(0%)	0(0%)	1(1%)	1.000
UTI	4 (5%)	2 (3%)	0 (0%)	6 (3%)	0.747 ^1^
**Acute Surgical Complications**					
Wound Infection	1 (1%)	1 (2%)	2 (8%)	4 (2%)	0.155 ^1^
Wound Dehiscence/Excess Wound Drainage	1 (1%)	0 (0%)	1 (4%)	2 (1%)	0.414 ^1^
Periprosthetic Fracture	0 (0%)	1 (2%)	0 (0%)	1 (1%)	0.520 ^1^
**Chronic Surgical Complications**					
Wound Infection	0 (0%)	0 (0%)	1 (4%)	1 (1%)	0.150 ^1^
Joint Infection	1 (1%)	0 (0%)	1 (4%)	2 (1%)	0.414 ^1^
Dislocation	2 (2%)	0 (0%)	1 (4%)	3 (2%)	0.275 ^1^
Periprosthetic Fracture	0 (0%)	2 (3%)	0 (0%)	2 (1%)	0.269 ^1^

^1^ Fisher’s Exact Test for Count Data. DVT: deep venous thrombosis, PE: pulmonary embolism, AFib: atrial fibrillation, MI: myocardial infarction, UTI: urinary tract infection.

**Table 4:** Reoperations and revisions.

	Kidney (N = 77)	Liver (N = 77)	Multiple (N = 22)	Total (N = 176)	*p* Value
**Revisions**	1 (1%)	0 (0%)	1 (4%)	2 (1%)	0.414 ^1^
**Reoperations (including Revisions)**	3 (4%)	0 (0%)	2 (8%)	5 (3%)	0.104 ^1^

^1^ Fisher’s Exact Test for Count Data.

## Data Availability

The data presented in this study are available on request from the corresponding author due to the institutional HIPAA compliant database from which the data are derived.
